# Capturing quality of life in pouch anal and vaginal fistula: development of PAVF-QoL-14, a patient-reported outcome measure

**DOI:** 10.1007/s11136-026-04380-z

**Published:** 2026-09-03

**Authors:** Lillian Reza, Laith Alrubaiy, Lara Bapir, Nusrat Iqbal, Charlene Sackitey, Easan Anand, Sally Hughes, Mina Babbar, Rali Marinova, Petya Marinova, Pearl Avery, Phillip Lung, Ailsa Hart, Sue Clark, Phil Tozer

**Affiliations:** 1 Robin Phillips Fistula Research Unit, St Mark’s Hospital, Imperial College London, London, UK; 2 IBD Unit, St Mark’s Hospital, London, UK; 3 West Middlesex University Hospital, London, UK; 4 Imperial College, London, UK; 5 Robin Phillips Fistula Research Unit, St Mark’s Hospital, London, UK; 6 St Mark’s Hospital, London, UK; 7 Crohn’s & Colitis UK, Hatfield, UK; 8 IBD Unit, St Mark’s Hospital, Imperial College, London, UK; 9 St Mark’s Hospital, Imperial College, London, UK

**Keywords:** Ileoanal pouch, Inflammatory bowel disease, Core outcome set, Patient reported outcomes

## Abstract

**Introduction:**

Perianal pouch fistula can significantly affect quality of life, yet no disease-specific patient reported outcome measure (PROM) exists. We have developed and validated a 14-item PROM—the Pouch Anal and Vaginal Fistula Quality of Life scale (PAVF-QoL-14).

**Methods:**

COnsensus-based Standards for the selection of health Measurement INstruments (COSMIN) guidance for PROM development was used to develop and validate PAVF-QoL-14. A systematic literature review and semi-structured patient interviews (n = 14) identified key domains. Cognitive interviews and item-level content validity index with experts refined the draft questionnaire. Psychometric evaluation included principal component analysis (PCA), internal consistency testing, and construct validity assessment against Hospital Anxiety and Depression Scale (HADS) and Short-Form 12 (SF-12). Test–retest analysis was performed on 76 patients.

**Results:**

An initial 34-item draft scale was generated and refined through cognitive interviews (n = 10) and psychometric testing (n = 76). Flesch readability score was 63%. Kaiser–Meyer–Olkin test confirmed sampling adequacy (KMO = 0.905), and correlations between items were found significant using Bartlett’s test of sphericity (*p* < 0.001). Four domains (symptoms, discharge, faecal incontinence, and future concerns) were extracted, explaining 63.7% of the total variance. Following stepwise regression, a 14-item short-form was developed, demonstrating excellent internal consistency (Cronbach’s α = 0.920). Responsiveness was good (effect size 0.77), and test–retest reliability demonstrated excellent stability (ICC = 0.924). Construct validity was supported by moderate-to-strong correlations with HADS (r = 0.589, *p* < 0.001) and SF-12 (r = 0.525, *p* < 0.001). The final questionnaire generates scores ranging from 14 to 70, with higher scores indicating greater fistula-related impairment in quality of life.

**Conclusion:**

PAVF-QoL-14 provides a concise, validated, and disease-specific measure of quality of life for pouch-related fistula and shows strong potential for use in clinical practice and clinical trials.

**Supplementary Information:**

The online version contains supplementary material available at https://doi.org/10.1007/s11136-026-04380-z.

## Abstract (plain language summary)

People who have an ileoanal pouch can sometimes develop a pouch anal or vaginal fistula, which is an abnormal connection that may cause ongoing discharge, pain, infections, and emotional distress. These symptoms can have a major impact on daily activities, relationships, work, and overall wellbeing. Despite this, there has been no questionnaire specifically designed to measure how pouch-related fistulas affect quality of life from the patient’s perspective.

In this study, we developed and tested a new questionnaire called the Pouch Anal and Vaginal Fistula Quality of Life questionnaire (PAVF-QoL-14). Patients with lived experience of pouch fistula were involved throughout the process, starting with interviews to identify the symptoms and concerns that mattered most to them. The questionnaire was then refined through patient feedback and expert review to ensure that the questions were clear, relevant, and easy to understand. We tested the questionnaire in a group of patients and showed that it is reliable, meaning people gave similar answers when their condition had not changed, and responsive, meaning scores changed appropriately when symptoms improved or worsened. The final questionnaire contains 14 questions covering areas such as symptoms, discharge, bowel control, tiredness, emotional wellbeing, intimacy, and worries about the future. PAVF-QoL-14 is a short, patient-centred tool that can be used in routine clinical care and in research studies to better understand the impact of pouch fistula and to assess whether treatments improve quality of life.

**Supplementary Information:**

The online version contains supplementary material available at https://doi.org/10.1007/s11136-026-04380-z.

## Introduction

The ileoanal pouch was developed with the aim of improving quality of life for patients requiring proctocolectomy [1–3]. Pouch fistula is a debilitating complication that can significantly impair quality of life (QoL) and is associated with an increased risk of pouch failure. A recent systematic review and meta-analysis reported a pooled prevalence of pouch fistula of approximately 5% in patients with ulcerative colitis following restorative proctocolectomy and ileal pouch-anal anastomosis [4]. Systematic reviews report an overall pouch failure rate of 10%. However, the development of fistula is associated with a substantial escalation in risk, with pouch failure rates rising to as high as 50% in selected tertiary referral cohorts managing complex pouch fistula disease [5]. Despite its clinical impact, research on pouch fistula remains limited, with few prospective studies evaluating patient-centred outcomes. Furthermore, no validated disease-specific patient-reported outcome measure (PROM) currently exists to assess the impact of pouch anal and vaginal fistula on quality of life. Recently, ‘ileoanal pouch syndrome’ was defined as the constellation of symptoms experienced following ileal pouch-anal anastomosis, leading to the development of the Ileoanal Pouch Syndrome Severity (IPSS) score to assess pouch function and its impact on quality of life [6]. Although patients with pouch fistula have higher IPSS scores than those without fistula, the IPSS was designed to measure overall pouch function rather than the specific physical, psychological and social burden associated with pouch anal and vaginal fistula [7]. Likewise, generic PROMs such as SF-12 are insufficiently sensitive to detect fistula-related changes, underscoring the need for a disease-specific measure [8]. To our knowledge, there is currently no validated disease-specific PROM for patients with pouch anal and vaginal fistula.

**Fig. 1:**
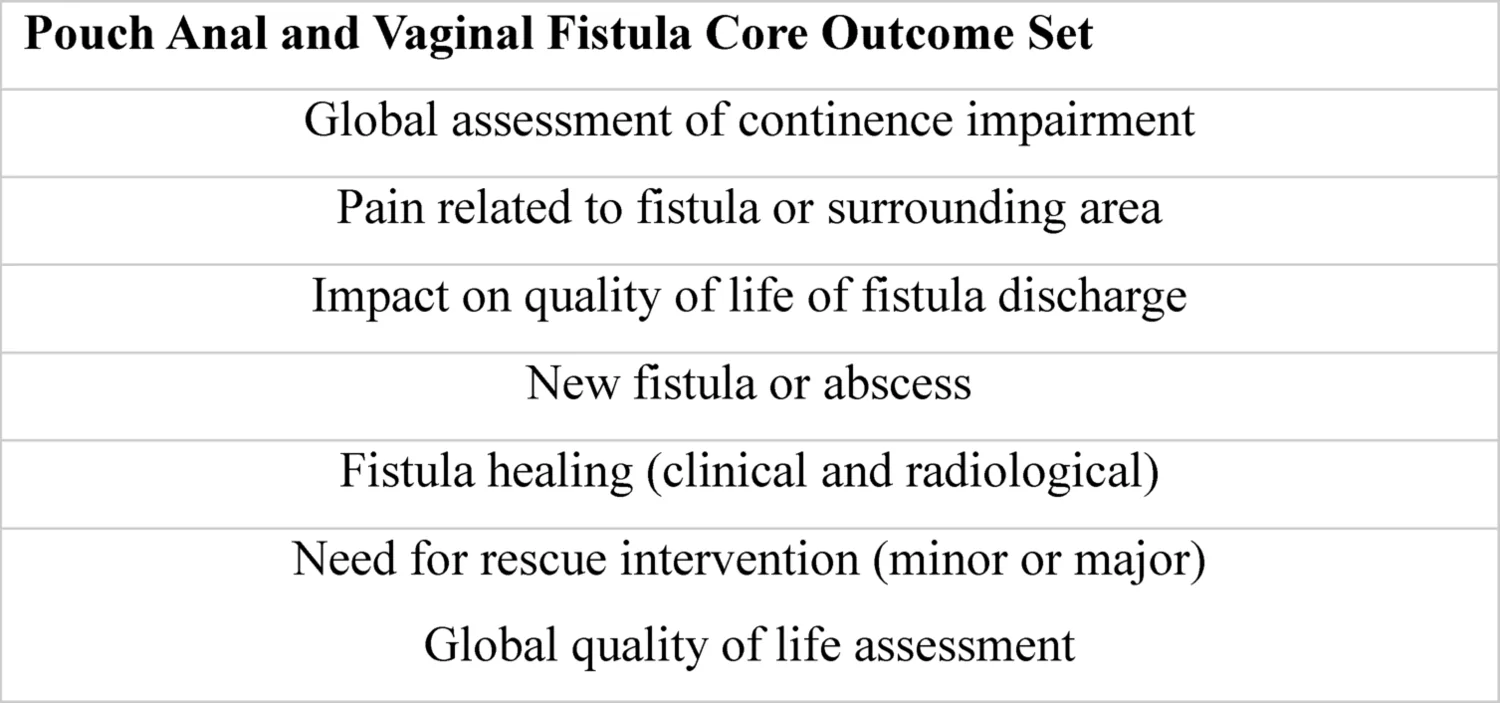
Pouch anal and vaginal fistula core outcome set

The pouch anal and vaginal fistula core outcome set (PAVFCOS) Delphi consensus study has established the core outcomes that should be measured in all interventional studies of pouch fistula, and this includes QoL outcomes such as global assessment of quality of life and impact on quality of life of fistula discharge (Fig. 1) [9]. This study aimed to develop a disease-specific QoL measurement tool in pouch anal and vaginal fistula (PAVF-QoL) that could be utilised for COS implementation in research and clinical assessment.

## Method

### Study design and reporting standards

The development of the PAVF-QoL scale followed the COnsensus-based Standards for the selection of health Measurement INstruments (COSMIN) taxonomy, which provides a structured approach for the development and evaluation of patient-reported outcome measures (PROMs) [10]. This study is reported in line with the COSMIN study design checklist for PROMs, ensuring transparency, methodological rigour, and adherence to best practice guidelines for outcome instrument development [11]. Ethical approval was granted by the Wales Research Ethics Committee 3 (REC reference: 20/WA/0308), and the Health Research Authority (HRA) and Health and Care Research Wales.

### Research aim

Our aim was to develop and validate a disease specific PROM to assess the impact of pouch anal and vaginal fistula symptoms, and their treatment, on health-related quality of life of patients with an ileoanal pouch.

### Study management group

The study was overseen by a multidisciplinary management group comprising patients and clinicians. Patients with lived experience of pouch-related fistula were involved from the outset to ensure that the instrument captured relevant aspects of their lived experience. The clinical members of the team included pouch dysfunction specialist colorectal surgeons and gastroenterologist, specialist pouch nurses, and a radiologist with expertise in pouch imaging. In addition, the group included clinicians and researchers with experience in PROM development and qualitative methodology. The study management group (SMG) guided each stage of the development process, from defining the construct, generating items, to reviewing content validity and refining the instrument. Patients contributed throughout, ensuring that themes reflected patient priorities rather than researcher assumptions.

### Definition of construct and conceptual framework

PAVF-QoL was developed to assess the impact of pouch-related perianal fistula and their treatments on the physical, psychological, and social functioning of patients with an ileoanal pouch. The construct being measured is health-related quality of life in chronic perineal fistulising disease in patients with an ileoanal pouch.

Instrument design was informed by Wilson and Cleary’s model of health-related quality of life, which provides a structured framework linking biological and physiological factors (e.g. the presence of a fistula) to symptoms, functional status, general health perceptions, and overall quality of life. This model was previously and successfully applied in the development of the Anal Fistula Quality of Life scale (AF-QoL), a disease-specific PROM for cryptoglandular anal fistula [12] and in the development of Crohn’s Anal Fistula Quality of Life (CAF-QoL) scale for patients with Crohn’s anal fistula [13]. Both highlighting the complex, multidimensional impact of fistula disease on quality of life. Patients with pouch-related fistula experience a similarly multifaceted burden but often with additional symptoms specific to pouch anatomy and function. Pouch-specific symptoms, including altered bowel habit, pouchitis, and pouch dysfunction required careful delineation and exclusion during item generation to ensure that the scale reflected the impact of the fistula and its treatment, not general pouch function or dysfunction [7]. By focusing specifically on fistula-related morbidity rather than general pouch function, PAVF-QoL aims to isolate the impact of chronic perineal sepsis, drainage, and surgical intervention on everyday life, intimacy, and psychological wellbeing.

### Target population

PAVF-QoL has been developed to assess QoL in patients with pouch-related perianal fistula arising from anastomotic leak, non-Crohn’s active inflammatory bowel disease (IBD), Crohn’s disease or cryptoglandular disease related aetiologies—as described in the literature [14]. The instrument is applicable regardless of fistula duration, complexity, or treatment stage, allowing its use across a broad spectrum of clinical presentations. PAVF-QoL has been designed to aid clinical assessment, measure impact of intervention and provide standardisation in outcome reporting in clinical research.

### Participant eligibility selection

Adults aged ≥ 18 years with a diagnosis of pouch-related anal or vaginal fistula were eligible to participate. Participants were required to have capacity to provide informed consent and be fluent in written and spoken English. The index diagnosis at restorative proctocolectomy was recorded and included all patients with a pouch regardless of index diagnosis (Ulcerative Colitis, Familial Adenomatous Polyposis, Indeterminate Colitis and Crohn’s disease). For Phases 1 (item generation) and 2 (cognitive interviews), patients with experience of a pouch anal or vaginal fistula within the preceding 12 months were eligible, irrespective of whether they had subsequently undergone pouch excision. For Phase 3 (psychometric validation), participants were required to have an active pouch anal or vaginal fistula within the preceding 6 months. Patients who had undergone pouch excision were excluded from Phase 3 to ensure that questionnaire responses reflected the impact of an existing pouch fistula. Defunctioning ileostomy in the presence of a pouch with history of a fistula in the preceding 6 months did not exclude patients from participating in any phase of the study as pouch fistulas may persist and be symptomatic despite defunctioning.

Purposive sampling recruitment was used to ensure representation of a broad range of experiences, fistula complexity, and chronicity. Primary recruitment was primarily conducted at a tertiary pouch centre. Patients were also recruited through the Red Lion pouch patient support group and social media. Recruitment (October 2022- August 2024) through patient groups and social media enabled inclusion of individuals receiving follow-up outside specialist centres, thereby improving the diversity and generalisability of the study.

The methodology for instrument development has been modelled on the study developing the AF-QoL scale [12] and this has been described according to three distinct phases.

### Phase 1: item generation

Item generation utilised inductive and deductive methods to ensure comprehensive content coverage and robust conceptual validity. Items were derived from semi-structured interviews with patients with pouch fistula. These semi-structured interviews were conducted using a topic guide (see supplementary file) by a researcher trained in qualitative interviews. Interviews were recorded and transcribed verbatim. The qualitative methodology has been described in detail in the development of the Pouch Anal and Vaginal Fistula Core Outcome Set (PAVFCOS), which was conducted in parallel with the present study [9]. Findings from the interviews were supplemented with a systematic review of existing PROMs used in studies of pouch fistula [8]. Interviews explored patients’ lived experiences of the condition, including its physical, psychological, and social impact, as well as the consequences of treatment. The interviews were analysed using thematic analysis to identify recurrent themes relating to the physical, psychological and social impact of pouch fistula. Emerging themes were reviewed by the study management group and mapped to the domains of Wilson and Cleary’s health-related quality of life model. These domains subsequently informed item generation and the conceptual framework underpinning the draft questionnaire. Patient representatives in the study management group ensured that verbatim responses from patient interviews were appropriately converted into items using patient derived language and reflected a distinct QoL construct. Items were mapped to physical symptoms, impact of symptoms on mental health, sex and intimacy, and global quality of life. Survey instructions, item wording and responses were developed with patient representatives to ensure readability, clinical relevance and acceptability. A recall period was set at 4–6 weeks to limit recall bias.

Flesch Reading Ease and Flesch–Kincaid grade level was assessed to determine readability. Statistical analysis was conducted using SPSS Version 29.

### Phase 2: scale development

#### Cognitive interviews

The draft scale was assessed for relevance, comprehensiveness and comprehensibility using cognitive interviews with patient participants. Participants completed the draft scale using the think-aloud technique and were probed for feedback using a predetermined probing questionnaire (see supplementary file). Interviews were conducted by a qualitative researcher, recorded and transcribed verbatim. Interviews were analysed to amend the draft questionnaire. Three consecutive interviews were analysed at a time until no further changes were suggested. The item level content validity index (I-CVI) was assessed by experts in pouch fistula using a Likert scale (1 = not at all relevant, 4 = extremely relevant) to rate the relevance of each item in the assessment of QoL. Items with an I-CVI < 0.78 were assessed for exclusion. The scale level content validity was calculated and a level > 0.90 demonstrated excellent content validity [10].

### Phase 3: psychometric analysis

The test phase recruited patient participants from clinics, social media and patient groups. The draft PAVF-QoL questionnaire was distributed with a demographic questionnaire, Short Form-12 (SF-12) [15], Hospital Anxiety and Depression Scale (HADS) [16] and the patient reported component of the Perianal Disease Activity Index (PDAI) [17]. For test–retest analysis, the questionnaire was resent with an interval of 4–6 weeks alongside a background form with questions to assess change in symptoms, disease severity and patient perceived impact on quality of life since last completion of PAVF-QoL (see supplementary file). The background form contained composite anchor questions which identified patients with stable disease and unstable disease. Psychometric analysis was performed to evaluate the validity, reliability, responsiveness, and interpretability of the PAVF-QoL [18, 19]. The complete questionnaire utilised in the test–retest phase is included in supplementary files.

### Internal consistency

Internal consistency of PAVF-QoL was measured using the Cronbach’s alpha statistic with an eigen > 0.7 deemed acceptable [20]. Item–total and item–item correlations (ITC and IIC) were examined at baseline (index completion of PAVF-QoL) to identify redundancy. Items showing excessive correlation with the total score or with other items were considered for exclusion to reduce duplication. Items with ITC <0.30 were flagged for weak association with the construct; pairs with IIC >0.80 were flagged as duplicative [20, 21].

### Factor structure

Principal factor analysis (PFA), using principal component extraction followed by Varimax rotation with Kaiser normalisation, was performed to explore the underlying domains of PAVF-QoL. Kaiser–Meyer–Olkin (KMO) test and Bartlett’s test of sphericity were applied to confirm factorability. Exploratory PCA is a statistical technique used to identify clusters of questions that measure similar constructs and explain the greatest variance within the scale. Domains were considered important if their corresponding eigen value exceeded 1 and they explained more than 5% of the total variance. Items were considered to contribute meaningfully to a domain if their loading was ≥0.4. Items not loading onto any important domain were considered for removal from the final instrument [20].

### Stepwise regression

Stepwise regression was performed as an item-reduction technique to identify the smallest subset of items that explained the greatest proportion of variance in the overall PAVF-QoL score. In addition, clinical relevance, patient-prioritised content and conceptual balance across domains were considered when determining the final items retained in PAVF-QoL-14 [22, 23].

### Construct validity

Construct validity measures how consistently a scale aligns with other validated scales assessing similar traits. Correlations between PAVF-QoL scores and recognised indicators of psychological wellbeing, symptom burden, and functional status were evaluated to investigate construct validity. Pearson’s correlation coefficients were calculated for PAVF-QoL in relation to the patient-reported scores from the Perianal Disease Activity Index (PDAI), Short Form-12 (SF-12), and the Hospital Anxiety and Depression Scale (HADS). Significant relationships are indicated by correlation coefficients ranging from 0.4 to 0.8 [19, 21, 24].

### Test–retest reliability

Test–retest reliability was assessed in participants reporting stable disease over a 4–6 week interval. Intraclass correlation coefficients (ICC), calculated using analysis of variance, were used as the primary measure of agreement between surveys and ICC >0.70 was considered acceptable. Pearson’s correlation coefficient was also calculated to assess the correlation between test and retest scores [19].

### Responsiveness

Responsiveness reflects the ability of the PAVF-QoL to detect clinical change. It was assessed in patients who reported improvement or deterioration in their fistula condition between assessments, in contrast to reliability which was tested in stable patients [20]. Three metrics were used:

- Responsiveness ratio (RR): mean change in scores of patients reporting improvement or deterioration, divided by the SD of baseline scores in stable patients [25].
- Effect size (ES): mean change in scores of patients reporting improvement or deterioration, divided by the SD of baseline scores in that same group. ES values of 0.2–0.49 were considered small, 0.5–0.79 moderate, and ≥ 0.8 large [25].
- Standardised response mean (SRM): mean change in scores of patients with improvement or deterioration, divided by the SD of the change scores between assessments. SRM >0.8 was considered very good [25, 26].

The retest sample was grouped by reported symptom status: no change, improvement, or worsening. Mean test–retest score differences with confidence intervals (CIs) were calculated and compared using paired t-tests, with significance set at *p* < 0.05.

### Interpretability

Patients completed a self-reported visual analogue score (VAS) rating how bothersome their fistula was on a scale of 1–10 (1 = not bothersome at all, 10 = extremely bothersome). The minimally important change (MIC) was defined as the mean change in PAVF-QoL score among patients who reported a 1–2 point change on the VAS. This represents the smallest difference in score perceived as important by patients, providing a threshold for interpreting whether changes reflect meaningful improvement or deterioration following intervention [12, 27].

## Results

### Study population

Across the three phases, 99 patients participated (Phase 1: *n *= 14; Phase 2: *n *= 9; Phase 3: *n *= 76). The majority of participants had undergone restorative proctocolectomy with ileal pouch-anal anastomosis for ulcerative colitis (80, 81%), while 6 (6%) had familial adenomatous polyposis, 4 (4%) had Crohn’s disease, and the underlying diagnosis was unknown in 9 (9%). In the psychometric testing cohort, two-thirds were female (*n *= 51, 67%) with a median age of 53 years (IQR 40–60). The majority had a disease duration > 48 months (*n *= 48, 63%), with a smaller proportion reporting 25–48 months (*n *= 15, 20%) or <24 months (*n *= 10, 13%). At the time of testing, 70% (*n *= 53) reported an active fistula, 26% (*n *= 20) were inactive, and 4% (*n *= 3) were unsure. Fistula type was anal in 54% (*n *= 41), vaginal in 28% (*n *= 21), and both anal and vaginal in 9% (*n *= 7), while 9% (*n *= 7) were unspecified (Table 1).

**Table 1:** Participant demographics in each phase

	Phase 1 Item generation (*N*=14), *n* (%)	Phase 2 Cognitive Interviews (*N*=9), *n* (%)	Phase 3 Psychometric Testing (N=76), *n* (%)
*Gender*			
Male	6 (43)	4 (44)	25 (33)
Female	9 (57)	5 (56)	51 (67)
Median age (years) (IQR)	47 (40–59)	41 (32–50)	53 (40–60) years
*Duration of diagnosis*			
0–24	5 (36)	2 (22)	10 (13)
25–48	5 (36)	4 (44)	15 (20)
>48	4 (28)	3 (34)	48 (63)
Unknown			3 (4)
*Active fistula*			
Yes	8 (57)	7 (78)	53 (70)
No	4 (29)	1 (11)	20 (26)
Not sure	2 (14)	1 (11)	3 (4)
*Fistula type*			
Anal	4 (29)	7 (78)	41(54)
Vaginal	6 (43)	2 (22)	21(28)
Both	3 (21)		7 (9)
Unknown			7 (9)
Stoma	1 (7)		14 (18)

### Phase 1: item generation

The study management group produced the first draft of PAVF-QoL with 32 items using themes generated from 14 patient interviews, and systematic review of the literature [9].

### Phase 2: scale development

#### Cognitive interviews

A total of 9 patients were interviewed over 3 rounds to revise wording of items and responses. Three additional questions were suggested with multiple revisions made to structure of questions and responses. The initial three cognitive interviews were on average 30 min in length while in the final round the interviews ranged between 5 and 7 min demonstrating improvement in structure and readability. Of the three new questions suggested, the SMG found one suggested question to be closely related to an existing item and the other two questions were included in the draft (see supplementary Table 1). Content validity was assessed by 8 experts and reported with the mean individual item rankings (I-CVI). The SMG decided to retain all items including the two items with an I-CVI < 0.78 as these were highly prioritised by patients in the interviews (see supplementary Table 2).

### Phase 3: psychometric testing

A total of 76 patients completed the draft PAVF-QoL with 34 items along with the validated questionnaires and background form. The second round was completed by 55 patients.

### Scoring of PAVF-QoL

The 14 items were each rated on a scale from 1 to 5. The total scores range from 14 to 70. For question 16 and 19, the “Not applicable” response is assigned a value of zero. Higher total scores indicate a greater impact of symptoms and worse quality of life.

### Item reduction

Table 2 demonstrates Item-item correlations. Items with ITC <0.30 and item-item correlations suggestive of redundancy were flagged for review but were not removed at this stage. All items were carried forward into principal factor analysis, where items with factor loadings ≥0.4 were considered to contribute meaningfully to an underlying domain, and subsequently into stepwise regression for item reduction. Final item retention was determined using statistical assessment together with clinical relevance, patient priorities identified during qualitative interviews, and conceptual representation across the four domains.

**Table 2:** Item–total correlations (ITC) for candidate PAVF-QoL items at baseline

Item-total correlations	
PQOL1= Do you get swelling around the anus/vagina because of your fistula?	0.712
PQOL2= How often do you experience discharge (pus/mucus/blood) from your fistula?	0.575
PQOL3= Do you need to use pads/gauze because of discharge from your fistula?	0.714
PQOL4= How much pain have you had around your anus/vagina because of the fistula in the past 6 weeks?	0.760
PQOL5= How often do you experience discomfort from your fistula?	0.748
PQOL6= Do you get skin irritation (chafing) because of discharge from your fistula?	0.749
PQOL7= How often do you need to use medication for pain from your fistula?	0.687
PQOL8= How often have you felt unwell because of your fistula in the past 6 weeks?	0.753
PQOL9= How often have you leaked stool through your fistula in the past 6 weeks?	0.744
PQOL10= How often do you use medication (e.g. Loperamide, codeine) to control leakage of stool through your fistula?	0.505
PQOL11= How often are you bothered by side effects of medications (loperamide, codeine, antibiotics, biologics, immunomodulators) used to treat your fistula?	0.522
PQOL12= I have to modify my diet to control the symptoms of my fistula	0.609
PQOL13= I feel tired or drained because of my fistula	0.849
PQOL14= I am worried others may be able to smell the discharge from my fistula	0.754
PQOL15= I worry about what others might think as I need to frequently use the toilet because of my fistula	0.720
PQOL16= I worry about becoming incontinent, or losing control of my pouch because of fistula surgery	0.282
PQOL17= I worry that I may need to have more surgery in the future for my fistula	0.608
PQOL18= I worry about getting another fistula or abscess	0.465
PQOL19= I worry about needing a stoma because of my fistula	0.376
PQOL20= I feel embarrassed about having a fistula	0.637
PQOL21= I feel sad/depressed because of my fistula	0.748
PQOL22= I worry about having children in the future as it may worsen my fistula symptoms	0.226
PQOL23= I worry about not being able to work, or needing to make changes to my job in the future due to my fistula (e.g. having to work from home)	0.640
PQOL24= Symptoms from my fistula (e.g. pain/discharge) affect my sexual activity	0.674
PQOl25= Symptoms from my fistula affect my ability or desire to get physically close/ be intimate with someone	0.640
PQOL26= How often has your sleep been disturbed because of your fistula?	0.830
PQOL27= How often is your ability to concentrate disturbed because of your fistula?	0.816
PQOL28= How often has the fistula limited your ability to work (household or work outside the home)?	0.807
PQOL29= How often has the fistula limited physical activities that you would like to do (swimming/exercise/running/cycling)?	0.814
PQOL30= How often does your fistula affect your choice of transportation (travelling by car, bus, train)?	0.730
PQOl31= I avoid attending social events because of my fistula	0.728
PQOL32= I have had to leave social events early because of my fistula	0.770
PQOL 33= I only attend social events if I know there will be toilets nearby because of my fistula	0.742
PQOL34= I feel supported by my family and friends to manage my fistula	− 0.151

### Principal factor analysis

Sampling adequacy was excellent (KMO = 0.905), and Bartlett’s test was highly significant (*p *< 0.001), indicating that the data were suitable for factor analysis. Initial analysis suggested nine potential domains, however applying standard criteria (eigenvalue >1, contribution to >5% of variance, and principal factor analysis in supplementary Table 3), supported retention of four key domains—‘symptoms’, ‘discharge’, ‘faecal incontinence’, and ‘future concerns’ that the items could be grouped in to. The symptoms domain captured pain, fatigue and limitations in daily activities; the discharge domain reflected the physical consequences of fistula discharge, including skin irritation and pad use; the faecal incontinence domain assessed leakage and concerns regarding continence; and the future concerns domain captured worries relating to further surgery, recurrent fistula and the long-term impact of the condition. Scree plot inspection demonstrated a marked elbow at the fourth domain, after which eigen values plateaued, supporting retention of four domains (supplementary Fig. 1). These four domains were extracted and rotated using Varimax with Kaiser normalization, explaining a cumulative 63.7% of the total variance. Items were considered meaningful if they loaded ≥0.4 on one of the four domains. The retained items were well distributed across the four domains, confirming the multidimensional structure of the PAVF-QoL. Table 3 demonstrates factor loadings of PAVF-QoL-14 items following Varimax rotation (Kaiser normalization). Supplementary Table 3 includes PFA and factor loadings of PAVF-QoL-34 items following Varimax rotation (Kaiser normalization).

**Table 3:** Factor loadings of PAVF-QoL-14 items following Varimax rotation (Kaiser normalization)

		Domains/PCA Factors			
Q	PAVF-QoL-14	Symptoms	Discharge	Faecal incontinence	Future concerns
	PQOL3= Do you need to use pads/gauze because of discharge from your fistula?		0.571	0.513	
	PQOL4= How much pain have you had around your anus/vagina because of the fistula in the past 6 weeks?	0.734			
	PQOL6= Do you get skin irritation (chafing) because of discharge from your fistula?	0.495	0.473		
	PQOL7= How often do you need to use medication for pain from your fistula?	0.678			
	PQOL9= How often have you leaked stool through your fistula in the past 6 weeks?		0.475	0.590	
	PQOL13= I feel tired or drained because of my fistula	0.657			
	PQOL16= I worry about becoming incontinent, or losing control of my pouch because of fistula surgery				0.894
	PQOL18= I worry about getting another fistula or abscess	0.475			0.466
	PQOL20= I feel embarrassed about having a fistula	0.411	0.512		
	PQOL24= Symptoms from my fistula (e.g. pain/discharge) affect my sexual activity		0.895		
	PQOL26= How often has your sleep been disturbed because of your fistula?	0.534	0.418	0.435	
	PQOL28= How often has the fistula limited your ability to work (household or work outside the home)?	0.809			
	PQOL29= How often has the fistula limited physical activities that you would like to do (swimming/exercise/running/cycling)?	0.677		0.381	
	PQOL32= I have had to leave social events early because of my fistula	0.584		0.473	

The items retained for PAVF-QoL 14 are bold.

### Stepwise regression

Stepwise regression identified 12 items that explained the greatest proportion of variance. Two additional items (PQOL3 and PQOL4) were retained following review by the study management group because of their high clinical relevance and importance to patients identified during qualitative interviews, resulting in the final 14-item questionnaire. Table 4 demonstrates stepwise regression analysis and comparison of the draft 34 and the final PAVF-QoL-14 scale.

**Table 4:**
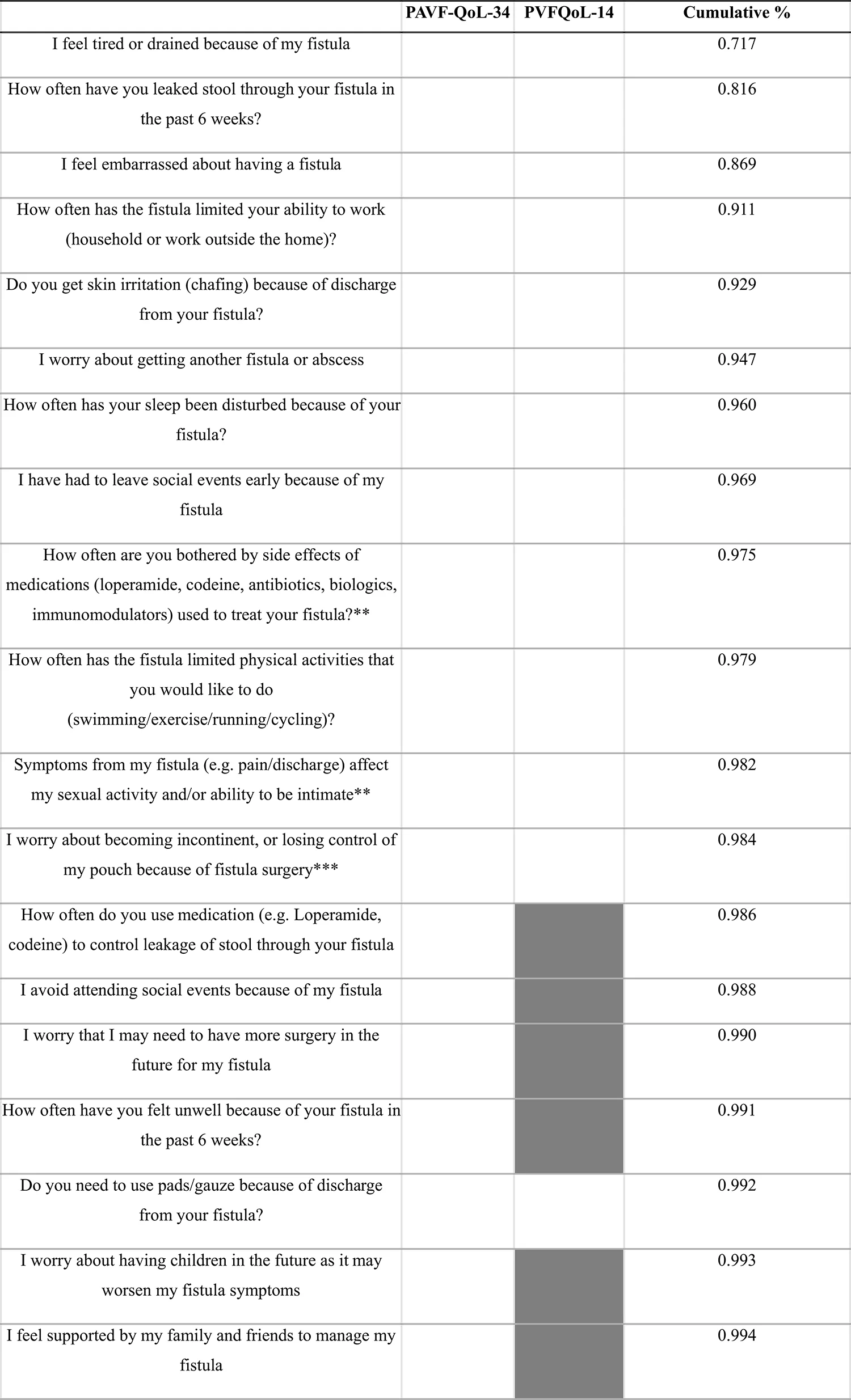

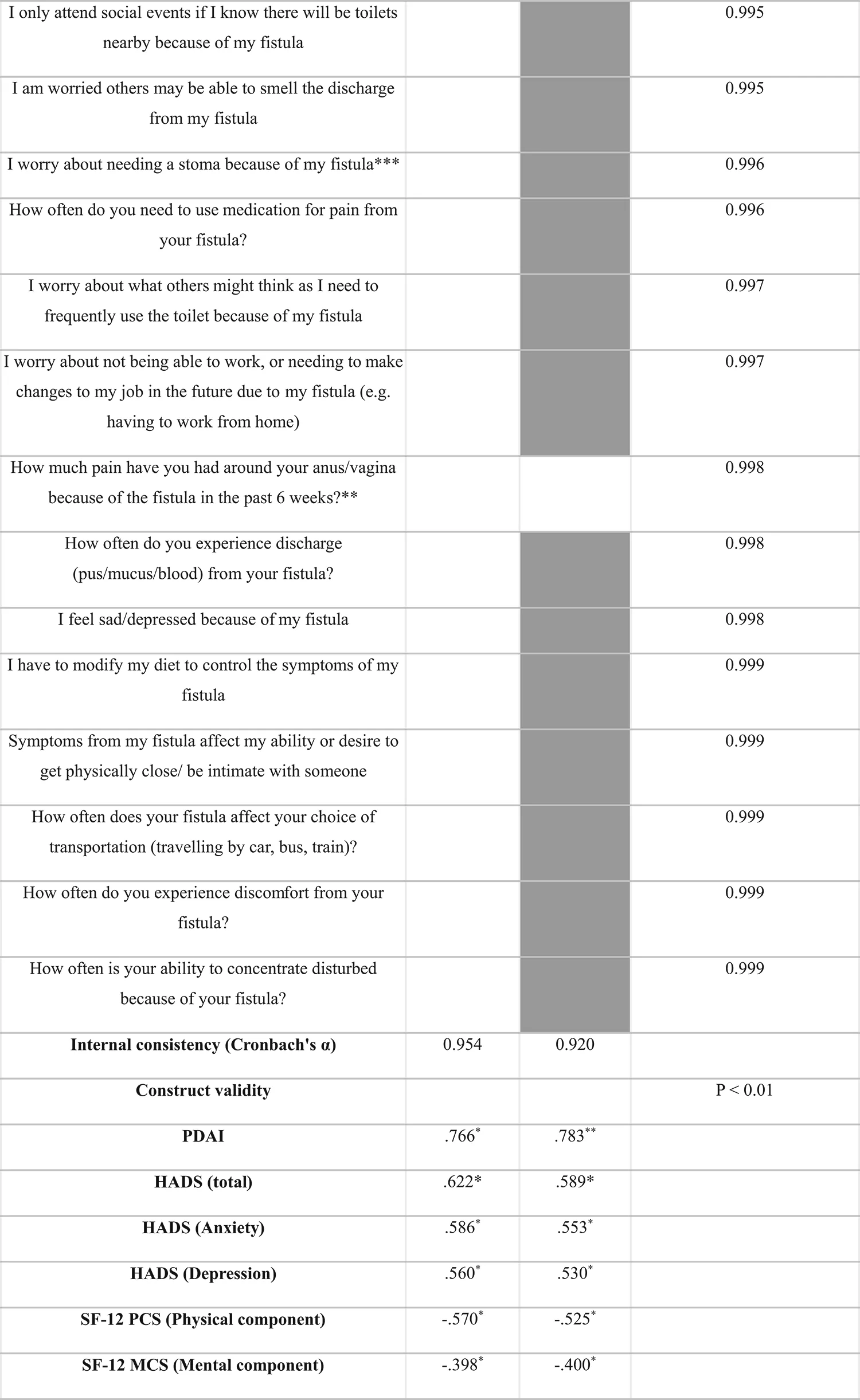
Stepwise regression analysis with comparison of PAVF-QoL 34 item and 14 item scale

Pearson correlation co-efficient*

Negative coefficients show that generic measures increase while disease specific measures decrease

Wording of item changed by SMG**

Questions with ‘not applicable’ as a possible response

Internal consistency remained very high (Cronbach’s α = 0.920 vs 0.954 for the full scale). Construct validity was maintained, with strong positive correlations with the PDAI (r = 0.783, *p *< 0.01) and HADS (r = 0.589, *p *< 0.01), and negative correlations with SF-12 physical and mental component scores (r = − 0.525 and − 0.400, *p *< 0.01 respectively). The final 14-item PAVF-QoL retained 63.7% of the variance explained by the original 34-item version, indicating that the shorter instrument captures most of the variability in patient responses.

### Test–retest reliability

Of the 55 patients who returned the retest questionnaire, 37 reported no change in symptoms and were included in reliability testing, while 18 reported a change and were included in sensitivity analyses. In the stable cohort, test–retest reliability was excellent, with intraclass correlation coefficients (ICC) of 0.943 for the full PAVF-QoL and 0.924 for the shortened PAVF-QoL-14 (both *p* < 0.01).

### Responsiveness

In patients reporting change (*n* = 18), both instruments demonstrated strong responsiveness. The responsiveness ratio (RR) was 0.643 for PAVF-QoL-34 and 0.639 for the PAVF-QoL-14. Effect sizes (ES) were 0.71 and 0.77, respectively, indicating moderate responsiveness. Standardised response means (SRM) were 0.945 for PAVF-QoL-34 and 1.071 for PAVF-QoL-14, both exceeding 0.8 and indicating very good responsiveness.

### Interpretability (MIC)

The MIC was 6 points for the original PAVF-QoL and 3 points for the PAVF-QoL-14, representing the smallest patient-perceived change considered clinically meaningful.

### PAVF-QoL-14

The final PAVF-QoL questionnaire consisted of 14 items and has a Flesch reading ease score of 67.4 and a Flesch-Kincaid grade level of 7.6 (reading age 12–14 years). This indicates that the questionnaire is written in plain English and is suitable for completion by the general adult population. The item reporting the impact of pain (PQOL 4) was modified to include discomfort in the wording as this reflects the scale of pain which could have varying degrees of impact on QoL. PQOL 10 wording was modified to reflect the burden of medication use to manage fistula. PQOL 24 includes ‘and/or ability to be intimate’ to ensure it reflects variation in experience. Item level content validity index (I-CVI) was reassessed by experts in pouch fistula for PAVF-QoL-14 and all items were rated > 0.80. Patients in the SMG agreed with the changes.

## Discussion

We have developed and validated a disease-specific PROM for pouch anal and vaginal fistula (PAVF-QoL-14) in accordance with COSMIN standards, addressing a longstanding measurement gap in pouch outcomes research. We derived items from qualitative interviews and the existing literature, and refined content through cognitive interviews and expert I-CVI assessment. Early and sustained patient involvement ensures face and content validity and produced an instrument that reflects patient prioritised outcomes.

Four-domains were identified using factor analysis (KMO 0.905; Bartlett *p *< 0.001) explaining 63.7% variance: symptoms, discharge, faecal incontinence, and future concerns. Stepwise regression reduced the 34-item draft to a user friendly 14-item tool without loss of measurement performance. Internal consistency remained excellent (α=0.920 vs 0.954), construct validity correlations with PDAI, HADS and SF-12 were preserved in magnitude and direction, and test–retest reliability was robust (ICC 0.924–0.943). Importantly, item retention decisions were not solely statistical: items with high patient relevance or clinical face validity were kept despite borderline statistics to maintain conceptual balance. The reduced form improved psychometric efficiency without sacrificing meaning. Responsiveness was acceptable to strong (RR≈0.64; ES 0.71–0.77; SRM 0.95–1.07), and anchor-based interpretability thresholds (MIC 6 points for the full scale; 3 points for the 14-item scale) provide an objective measure of clinical change.

PAVF-QoL-14 is a patient-centred tool designed to measure the multidimensional impact of pouch fistula and is concise for application in both clinical practice and research. It complements existing pouch function tools (e.g. IPSS) by isolating fistula-specific morbidity and its psychosocial impact, where symptoms from pouch function and fistula may otherwise be conflated [7]. Prior pouch literature leans on generic PROMs or non-validated symptom checklists, limiting comparability and effect-size detection. PAVF-QoL- 14 advances the field by (i) focusing on fistula-specific lived burden, (ii) reporting transparent scoring and (iii) demonstrating responsiveness to clinical change.

PAVF-QoL-14 includes psychometric evaluation of structure, reliability, validity, responsiveness, and interpretability. The four domains of PAVF-QoL identified using exploratory principal factor analysis reflects the core outcomes established in the PAVF core outcome set—pain related to fistula, global assessment of incontinence, fistula discharge, new fistula or abscess and need for rescue intervention [9]. PAVF-QoL-14 is a suitable instrument for global quality of life assessment thus enabling COS implementation.

There were limitations and challenges in validating a disease specific QoL scale in patients with pouch fistula. Patient recruitment proved challenging as anal and vaginal fistula are rare in the context of ileoanal pouch, occurring in 5% of an already small patient cohort. Although there is no set guidance on the number of patients required to validate a QoL scale, there is evidence that 5–10 patient responses may be required per item to validate the item and ensure it accurately reflects impact on QoL [28, 29]. The number of participants (n=76) in the test phase is sufficient to validate 14 items and KMO 0.905; Bartlett *p *< 0.001 analysis suggests that there were adequate participants to perform psychometric analysis of the scale.

Patients were mostly recruited from a single-centre, tertiary service which may limit generalisability but conversely a sufficient cohort of patients with pouch fistula could only be found at a referral centre specialising in complex pouch complications, and this rare and complex problem should generally be managed in a such centres, with experience of pouch dysfunction and complex fistula. Test–retest reliability was assessed with a 4–6 week gap to minimise recall bias while acknowledging that some patients may change clinically over this interval, which can attenuate reliability estimates.

### Future direction

Future work should focus on multicentre external validation, cross-cultural adaptation, longitudinal evaluation across treatment stages, and incorporation of PAVF-QoL-14 into core outcome measurement sets and prospective clinical trials. PAVF-QOL-14 could be adapted for electronic administration, which may enable effortless, longitudinal outcome collection. Formal electronic format validation and usability testing will be important next steps for digital implementation.

## Conclusion

PAVF-QoL-14 is a validated PROM specifically designed for pouch-related fistula, demonstrating strong psychometric properties. It is a concise, conceptually clear instrument which provides a disease-specific measure of global quality of life. This supports implementation of the Pouch Anal and Vaginal Fistula Core Outcome Set and may improve patient-centred outcome assessment in future clinical studies. Its immediate application is likely to be greatest in research and longitudinal follow-up within specialist referral centres, while external multicentre validation will further establish its wider clinical applicability.

## Supplementary Information

Below is the link to the electronic supplementary material.

Supplementary Material 1

Supplementary Material 2

Supplementary Material 3

Supplementary Material 4

Supplementary Material 5

Supplementary Material 6

## Data Availability

All data supporting the findings of this study are available within the paper and its Supplementary Information.
