# Supplementary material for: Capturing quality of life in pouch anal and vaginal fistula: development of PAVF-QoL-14, a patient-reported outcome measure
Source: Qual Life Res. 2026 Sep 3;35(10):279. doi: 10.1007/s11136-026-04380-z (PMC13541871; doi:10.1007/s11136-026-04380-z)
Supplement: Supplementary file 1 [file 11136_2026_4380_MOESM1_ESM.pdf]

PAVFQOL: Pouch Anal and Vaginal Fistula Quality of Life questionnaire

The aim of this questionnaire is to understand the impact of your fistula on your quality of life. Please answer the following questions focusing on your fistula symptoms and how they have affected your life over the last 4–6 weeks. If you have more than one fistula, consider the fistula or fistulas that cause you the most issues. Please answer all the questions. If you are unsure about how to answer any question, just give the best answer you can. Do not spend too much time thinking about your answer, as your first thoughts are likely to be the most accurate. If you do not wish to answer any of these questions, please leave it blank. Please leave details of the reason(s) why it was not answered.

Part 1: Your symptoms over the last 4–6 weeks

1. How often have you leaked stool through your fistula in the past 4–6 weeks?
   a) Never
   b) A little of the time
   c) Some of the time
   d) Most of the time
   e) All the time

2. Do you need to use pads/gauze because of discharge from your fistula?
   a) Never
   b) Occasionally
   c) One or more times a week
   d) Daily
   e) Several times a day

3. Do you get skin irritation (chafing) because of discharge from your fistula?
   a) None
   b) Very mild
   c) Mild
   d) Moderate
   e) Severe

4. How often have you had pain or discomfort around your anus/vagina because of the fistula in the past 4–6 weeks?
   a) Never
   b) A little of the time
   c) Some of the time
   d) Most of the time
   e) All the time

5. How often have you had to use medications (loperamide, codeine, antibiotics, biologics, immunomodulators) to manage your fistula?
   a) Never
   b) A little of the time
   c) Some of the time
   d) Most of the time
   e) All the time

Part 2: Daily life and physical activities

To what extent do you agree with the following statements:

6. I feel tired or drained because of my fistula
   a) Not at all
   b) Not much
   c) Somewhat/ a bit
   d) Quite a lot
   e) A great deal

7. How often has the fistula limited your ability to work (household or work outside the home)?
   a) Never
   b) A little of the time
   c) Some of the time
   d) Most of the time
   e) Always

8. How often has the fistula limited physical activities that you would like to do (e.g. exercise/sport)?
   a) Never
   b) A little of the time
   c) Some of the time
   d) Most of the time
   e) Always

9. How often has your sleep been disturbed because of your fistula?
   a) Never
   b) Occasionally
   c) One or more times a week
   d) Every night
   e) Several times a night

Part 3: Psychological wellbeing and future concerns

To what extent do you agree with the following statements:

10. I feel embarrassed about having a fistula
    a) Strongly disagree
    b) Disagree
    c) Neither agree nor disagree
    d) Agree
    e) Strongly agree

11. I worry about becoming incontinent, or losing control of my pouch because of fistula surgery
    a) Strongly disagree
    b) Disagree
    c) Neither agree nor disagree
    d) Agree
    e) Strongly agree
    f) Not applicable (I have a stoma)

12. I worry about getting another fistula or abscess
    a) Strongly disagree
    b) Disagree
    c) Neither agree nor disagree
    d) Agree
    e) Strongly agree

Part 4: Sex and intimacy

To what extent do you agree with the following statement:

13. Symptoms from my fistula, such as pain or discharge, impact my sexual activity and/or intimacy
    a) Not at all
    b) Rarely
    c) Some of the time
    d) Most of the time
    e) Always

Part 5: Social function

To what extent do you agree with the following statement:

14. I have had to leave social events early because of my fistula
    a) Strongly disagree
    b) Disagree
    c) Neither agree nor disagree
    d) Agree
    e) Strongly agree
