# Supplementary material for: Capturing quality of life in pouch anal and vaginal fistula: development of PAVF-QoL-14, a patient-reported outcome measure
Source: Qual Life Res. 2026 Sep 3;35(10):279. doi: 10.1007/s11136-026-04380-z (PMC13541871; doi:10.1007/s11136-026-04380-z)
Supplement: Supplementary file 2 [file 11136_2026_4380_MOESM3_ESM.pdf]

Demographics Form

DF1. Demorgraphics Form

The following questions are to gather some information about yourself and the history of your pouch fistula.

Unique ID number

DF2. Age

DF3. Ethnic Group (Please specify)

DF4. What year was your pouch created?

DF5. Why was your bowel removed and your pouch created?

- Ulcerative colitis
- Crohn's disease
- Familial adenomatous polyposis (FAP)
- Cancer
- Other
- Unknown

DF6. When were you diagnosed with a pouch fistula? (Year)

DF7. Please select your diagnosis if known to you:

- Pouch anal fistula
- Pouch vaginal fistula
- Both
- Not known

DF8. What is the cause of your fistula?

- Crohns disease
- Anastomotic leak following pouch operation
- Uknown
- Other (please specify)

DF9. Do you currently have an active fistula?

- Yes
- No
- Not sure

DF10. Do you currently have a stoma?

- Yes, due to fistula
- Yes, due to other reason
- No

DF11. Have you had an operation to remove your pouch?

- No
- [ ] Yes (please specify year pouch removed)

DF12. How many times has your fistula gotten worse in the last year (requiring surgical or medical attention)?

- 0 times
- 1-4 times
- 5 times or more
- Unsure

DF13. Do you have Crohn's Disease?

- Yes
- No
- Under investigation

DF14. If you have Crohn's disease, what disease medication do you currently take?

- ☐ Infliximab
- ☐ Adalimumab
- ☐ Azathioprne/Mercaptopurine
- ☐ Vedolizumab
- ☐ Ustekinimab
- ☐ None
- ☐ ____ Other (please specify)
- ☐ N/A

DF15. How many operations have you had on your fistula in total?

- ○ 0
- ○ 1
- ○ 2
- ○ 3
- ○ 4
- ○ 5
- ○ More than 5

DF16. What operations have you had for your fistula? (Tick all that apply)

- ☐ Seton insertion
- ☐ Laying open of fistula
- ☐ Perineal pouch advancement
- ☐ Transvaginal repair
- ☐ Glue/plug
- ☐ Redo pouch operation
- ☐ FILAC/LIFT
- ☐ Other (please specify)
- ☐ Unknown
- ☐ Not had any operations for fistula

Background Form

BF1. The following questions are to gather some information about how you feel about your fistula and the symptoms you are experiencing.

Overall how bothered are you about your fistula?

- ○ 0 (Not at all bothered)
- ○ 1
- ○ 2
- ○ 3
- ○ 4
- ○ 5

- ○ 6
- ○ 7
- ○ 8
- ○ 9
- ○ 10 (Extremely bothered)

BF2. Have your symptoms from your fistula changed since you last filled out a PAVF-QoL questionnaire? (only answer this question if you have filled out a questionnaire before)

- ○ I have not filled out this questionnaire before
- ○ Better
- ○ Worse
- ○ The same
- ○ Can't remember

BF3. What symptoms do you get with your fistula (tick all that apply)?

- ☐ Wind through the vaginal fistula
- ☐ Stool through the vaginal fistula
- ☐ Wind through anal fistula
- ☐ Stool through anal fistula
- ☐ Urinary tract infections
- ☐ Pain
- ☐ Smelly discharge

- ☐ Painful sex
- ☐ Sexual avoidance
- ☐ Low self-esteem / loss of self confidence
- ☐ Abscess formation

BF4. Has the number of times that you need to empty your pouch changed since you last completed the PAVF-QoL questionnaire? (only answer this question if you have filled out a questionnaire before)

- ○ I have not filled this questionnaire before
- ○ Better
- ○ Worse
- ○ The same
- ○ Can't remember

BF5. Do you find that a change in your pouch function (the number of times that you need to empty your pouch) affects symptoms from your fistula?

- ○ Yes
- ○ No
- ○ Unsure

BF6. How often do you forget about your fistula?

- Never
- Occasionally
- One or more times a week
- Daily
- Several times a day

PDAI

PDAI 1. **PERIANAL DISEASE ACTIVITY INDEX (PDAI)**

Please select the one that best describes living with a fistula

DISCHARGE

- No discharge
- Minimal mucous discharge
- Moderate mucous or purulent discharge
- Substantial discharge
- Gross faecal soiling

PDAI 2. PAIN/RESTRICTION OF ACTIVITIES

- No activity restriction

- Mild discomfort, no restriction
- Moderate discomfort, some limitation of activities
- Marked discomfort, marked limitation
- Severe pain, severe limitation

PDAI 3. RESTRICTION OF SEXUAL ACTIVITY

- No restriction of sexual activity
- Slight restriction of sexual activity
- Moderate limitation of sexual activity
- Marked limitation off sexual activity
- Unable to engage in sexual activity

HADS

HADS 1. **HOSPITAL ANXIETY & DEPRESSION SCALE (HADS)**

Please read each item below and select the box that comes closest to how you have been feeling in the past week

Tick one box per question

I feel tense and 'wound up'

- Most of the time
- A lot of the time

- Occasionally
- Not at all

HADS 2. **I still enjoy the things I used to enjoy**

- Definitely as much
- Not quite so much
- Only a little
- Hardly at all

HADS 3. **I get a sort of frightened feeling as if something awful is about to happen**

- Very definitely and quite badly
- Yes, but not too badly
- A little, but it doesn't worry me
- Not at all

HADS 4. **I can laugh and see the funny side of things**

- As much as always
- Not quite so much
- Definitely not so much now
- Not at all

HADS 5. **Worrying thoughts go through my head**

- A great deal of the time
- A lot of the time
- Not too often
- Very little

HADS 6. **I feel cheerful**

- Never
- Not often
- Sometimes
- Most of the time

HADS 7. **I can sit at ease and feel relaxed**

- Definitely
- Usually
- Not often
- Not at all

HADS 8. **I feel as if I am slowed down**

- Nearly all the time
- Very often
- Sometimes
- Not at all

HADS 9. **I get a sort of frightened feeling like ‘butterflies’ in my stomach**

- Not at all
- Occasionally
- Quite often
- Very often

HADS 10. **I have lost interest in my appearance**

- Definitely
- I don’t take as much care as I should
- I may not take as much care
- I take just as much care as ever

HADS 11. **I feel restless as if I have to be on the move**

- Very much indeed
- Quite a lot
- Not very much

- Not at all

HADS 12. **I look forward with enjoyment to things**

- As much as I ever did
- Rather less than I used to
- Definitely less than I used to
- Hardly at all

HADS 13. **I can enjoy a good book or radio or TV programme**

- Often
- Sometimes
- Not often
- Very seldom

HADS 14 . I get sudden feelings of panic

- Very often indeed
- Quite often
- Not very often
- Not at all

SF-12

SF12-1. **Short Form 12 Health Survey**

This survey asks for your views about your health. This information will help keep track of how you feel and how well you are able to do your usual activities. Answer each question by choosing just one answer. If you are unsure how to answer a question, please give the best answer you can.

In general, would you say your health is:

- Excellent
- Very good
- Good
- Fair
- Poor

SF12-2. The following questions are about activities you might do during a typical day. Does your health now limit you in these activities? If so, how much?

Moderate activities such as moving a table, pushing a vacuum cleaner, bowling or playing golf

- YES, limited a lot

- YES, limited a little
- NO, not limited at all

SF12-3. Climbing several flights of stairs

- YES, limited a lot
- YES, limited a little
- NO, not limited at all

SF12-4. During the **past 4 weeks,** have you had any of the following problems with your work or other regular daily activities **as a result of your physical health?**

Accomplished less than you would like.

- YES
- NO

SF12-5. Were limited in the **kind** of work or other activities

- YES
- NO

SF12-6. During the **past 4 weeks,** have you had any of the following problems with your work or other regular daily activities **as a result of any emotional problems** (such as feeling depressed or anxious)?

Accomplished less than you would like.

- YES
- No

SF12-7. Did work or activities **less carefully than usual.**

- YES
- NO

SF12-8. During the **past 4 weeks,** how much **did pain interfere** with your normal work (including work outside the home and housework)?

- None at all
- A little bit
- Moderately
- Quite a bit
- Extremely

SF12-9. These questions are about how you have been feeling during **the past 4 weeks.** For each question, please select the one answer that comes closest to the way you have been feeling.

How much of the time during the past 4 weeks…

Have you felt calm & peaceful?

- All of the time
- Most of the time
- A good bit of the time
- Some of the time
- A little of the time
- None of the time

SF12-10. **Did you have a lot of energy?**

- All of the time
- Most of the time
- A good bit of the time
- Some of the time
- A little of the time
- None of the time

SF12-11. **Have you felt down-hearted and blue?**

- ○ All of the time
- ○ Most of the time
- ○ A good bit of the time
- ○ Some of the time
- ○ A little of the time
- ○ None of the time

SF12-12. During the **past 4 weeks**, how much of the time has your **physical health or emotional problems** interfered with your social activities (like visiting friends, relatives, etc.)?

- ○ All of the time
- ○ Most of the time
- ○ A good bit of the time
- ○ Some of the time
- ○ A little of the time
- ○ None of the time

Draft PAVF-QoL

Q1.

Draft PAVFQoL: Pouch anal and vaginal fistula Quality of Life scale

The aim of this questionnaire is to understand the impact of your fistula on your quality of life. Please answer the questions focusing on your fistula symptoms and how these symptoms have **affected your life over the last 4-6 weeks.**

If you have more than one fistula, consider the fistula or fistulas, that cause you the most issues. Please answer all the questions. If you are unsure about how to answer any question, just give the best answer you can. Do not spend too much time thinking about your answer, as your first thoughts are likely to be the most accurate. If you do not wish to answer any of these questions, please leave it blank. Please leave details of the reason(s) why it was not answered.

Part 1: Your symptoms over the last 4-6 weeks

Do you get swelling around the anus/vagina because of your fistula?

- ○ Never
- ○ Occasionally
- ○ One or more times a week
- ○ Daily
- ○ Several times a day

Q2. How often do you experience discharge (pus/mucus/blood) from your fistula?

- Never
- Occasionally
- One or more times a week
- Daily
- Several times a day

Q3. Do you need to use pads/gauze because of discharge from your fistula?

- Never
- Occasionally
- One or more times a week
- Daily
- Several times a day

Q4. How much pain have you had around your anus/vagina because of the fistula in the past 6 weeks?

- None
- Very mild
- Mild
- Moderate

- Severe
- Very severe

Q5. How often do you experience discomfort from your fistula?

- Never
- Occasionally
- One or more times a week
- Daily
- Several times a day

Q6. Do you get skin irritation (chaffing) because of discharge from your fistula?

- None
- Very mild
- Mild
- Moderate
- Severe

Q7. How often do you need to use medication for pain from your fistula?

- Never

- Occasionally
- One or more times a week
- Daily
- Several times a day

Q8. How often have you felt unwell because of your fistula in the past 6 weeks?

- Never
- Occasionally
- One or more times a week
- Daily
- Several times a day

Q9. How often have you leaked stool through your fistula in the past 6 weeks?

- Never
- A little of the time
- Some of the time
- Most of the time
- All the time

Q10. How often do you use medication (e.g. Loperamide,

codeine) to control leakage of stool through your fistula?

- Never
- Occasionally
- One or more times a week
- Daily
- Several times a day

Q11. How often are you bothered by side effects of medications (loperamide, codeine, antibiotics, biologics, immunomodulators) used to treat your fistula?

- Never
- A little of time
- Some of the time
- Most of the time
- All the time

Q12. ***To what extent do you agree with the following statements?***

I have to modify my diet to control the symptoms of my fistula

- Not at all
- Not much
- Somewhat/ a bit

- Quite a lot
- A great deal

Q13. I feel tired or drained because of my fistula

- Not at all
- Not much
- Somewhat/ a bit
- Quite a lot
- A great deal

Q14. **Part 2: Impact of fistula on mental health in the last 4-6 weeks**

To what extent do you agree with the following statements?

I am worried others may be able to smell the discharge from my fistula

- Strongly disagree
- Disagree
- Neither agree nor disagree
- Agree
- Strongly agree

Q15. I worry about what others might think as I need to frequently use the toilet because of my fistula

- Strongly disagree
- Disagree
- Neither agree nor disagree
- Agree
- Strongly agree

Q16. I worry about becoming incontinent, or losing control of my pouch because of fistula surgery

- Strongly disagree
- Disagree
- Neither agree nor disagree
- Agree
- Strongly agree
- N/A (I have a stoma)

Q17. I worry that I may need to have more surgery in the future for my fistula

- Strongly disagree
- Disagree

- Neither agree nor disagree
- Agree
- Strongly agree

Q18. I worry about getting another fistula or abscess

- Strongly disagree
- Disagree
- Neither agree nor disagree
- Agree
- Strongly agree

Q19. I worry about needing a stoma because of my fistula

- Strongly disagree
- Disagree
- Neither agree nor disagree
- Agree
- Strongly agree
- N/A (I have a stoma)

Q20. I feel embarrassed about having a fistula

- Strongly disagree
- Disagree

- Neither agree nor disagree
- Agree
- Strongly agree

Q21. I feel sad/depressed because of my fistula

- Strongly disagree
- Disagree
- Neither agree nor disagree
- Agree
- Strongly agree

Q22. I worry about having children in the future as it may worsen my fistula symptoms

- Strongly disagree
- Disagree
- Neither agree nor disagree
- Agree
- Strongly agree

Q23. I worry about not being able to work, or needing to make changes to my job in the future due to my fistula (e.g. having to work from home)

- Strongly disagree
- Disagree
- Neither agree nor disagree
- Agree
- Strongly agree

Q24. **Part 3: Impact of fistula on sex and intimacy in the last 4-6 weeks**
To what extent do you agree with the following statements?

Symptoms from my fistula (e.g. pain/discharge) affect my sexual activity

- Not at all
- Rarely
- Some of the time
- Most of the time
- Always

Q25. Symptoms from my fistula affect my ability or desire to get physically close/ be intimate with someone

- Not at all
- Rarely
- Some of the time
- Most of the time

- Always

Q26. **Part 4: Impact of fistula on quality of life in the last 4-6 weeks**

How often has your sleep been disturbed because of your fistula?

- Never
- Occassionally
- One or more times a week
- Every night
- Several times a night

Q27. How often is your ability to concentrate disturbed because of your fistula?

- Never
- A little of the time
- Some of the time
- Most of the time
- Always

Q28. How often has the fistula limited your ability to work

(household or work outside the home)?

- Never
- A little of the time
- Some of the time
- Most of the time
- Always

Q29. How often has the fistula limited physical activities that you would like to do (swimming/exercise/running/cycling)?

- Never
- A little of the time
- Some of the time
- Most of the time
- Always

Q30. How often does your fistula affect your choice of transportation (travelling by car, bus, train)?

- Never
- A little of the time
- Some of the time
- Most of the time
- Always

Q31. **To what extent do you agree with the following statements**

I avoid attending social events because of my fistula

- Strongly disagree
- Disagree
- Neither agree nor disagree
- Agree
- Strongly agree

Q32. I have had to leave social events early because of my fistula

- Strongly disagree
- Disagree
- Neither agree nor disagree
- Agree
- Strongly agree

Q33. I only attend social events if I know there will be

toilets nearby because of my fistula

- Strongly disagree
- Disagree
- Neither agree nor disagree
- Agree
- Strongly agree

Q34. I feel supported by my family and friends to manage my fistula

- Strongly disagree
- Disagree
- Neither agree nor disagree
- Agree
- Strongly agree

Q35 . Comments

Accessibility

Powered by Qualtrics
