# Supplementary material for: Capturing quality of life in pouch anal and vaginal fistula: development of PAVF-QoL-14, a patient-reported outcome measure
Source: Qual Life Res. 2026 Sep 3;35(10):279. doi: 10.1007/s11136-026-04380-z (PMC13541871; doi:10.1007/s11136-026-04380-z)
Supplement: Supplementary file 3 [file 11136_2026_4380_MOESM2_ESM.docx]

**Developing a pouch anal and vaginal fistula quality of life scale (PAVF-QoL)**

**Guide for Cognitive Interviews**

**Introduction and consent**

a. Outline the purpose of the interview
b. Ensure patient still willing to take part
c. Check if participant agrees that the interview be audio-recorded, stored and used for presenting research findings anonymously.
d. Check if there are any questions regarding the interview procedure
e. Ask participant to complete the informed consent form

*Provide patient with the questionnaire and ask them to read out the questions while ‘thinking aloud’ their thoughts about them.*

**Probing Questions:**

1. Are the instructions understandable?
2. Are there any questions that you found difficult to understand?
3. Are there any questions you didn’t want to answer?
4. What do you think about the response options to the questions?
5. Do we need to add any additional questions?
6. What do you think about the recall period?
7. Are questions relevant to assessing the impact of your fistula on your quality of life?
