# Supplementary material for: Capturing quality of life in pouch anal and vaginal fistula: development of PAVF-QoL-14, a patient-reported outcome measure
Source: Qual Life Res. 2026 Sep 3;35(10):279. doi: 10.1007/s11136-026-04380-z (PMC13541871; doi:10.1007/s11136-026-04380-z)
Supplement: Supplementary file 5 [file 11136_2026_4380_MOESM5_ESM.docx]

**Supplementary Tables**

**Supplementary Table 1 Additional questions suggested during Cognitive Interviews**

| **Question** | **SMG Decision** |
| --- | --- |
| I feel supported by my family and friends to manage my fistula  a) Strongly disagree  b) Disagree  c) Neither agree nor disagree  d) Agree  e) Strongly agree | Include in PAVF QoL test questionnaire |
| I worry about not being able to work, or needing to make changes to my job in the future due to my fistula  a) Strongly disagree  b) Disagree  c) Neither agree nor disagree  d) Agree  e) Strongly agree | Include in PAVF QoL test questionnaire |
| I worry about having accidents in public owing to the fistula  a) Strongly disagree  b) Disagree  c) Neither agree nor disagree  d) Agree  e) Strongly agree | Do not include as already covered by an existing question |

**Supplementary Table 2 Content validity scored by 8 experts(I-CVI) for the 34 item scale**

| **Item** | **I -CVI** |
| --- | --- |
| Do you get swelling around the anus/vagina because of your fistula? | 1 |
| How often do you experience discharge (pus/mucus/blood) from your fistula? | 1 |
| Do you need to use pads/gauze because of discharge from your fistula? | 1 |
| How much pain have you had around your anus/vagina because of your fistula in the past 6 weeks? | 1 |
| How often do you experience discomfort from your fistula? | 1 |
| Do you get skin irritation (chaffing) because of discharge from your fistula? | 1 |
| How often do you need to use medication for pain from your fistula? | 0.87 |
| How often have you felt unwell because of your fistula in the past 6 weeks? | 0.87 |
| How often have you leaked stool through your fistula in the past 6 weeks? | 1 |
| How often do you use medication (e.g. Loperamide) to control leakage of stool through your fistula? | 0.87 |
| How often are you bothered by side effects of medications (loperamide, codeine, antibiotics, biologics, immunomodulators) used to treat your fistula? | 0.62 |
| I have to modify my diet to control the symptoms of my fistula | 0.87 |
| I feel tired or drained because of my fistula | 1 |
| I am worried others may be able to smell the discharge from my fistula | 1 |
| I worry about what others might think as I need to frequently use the toilet owing to my fistula | 0.87 |
| I worry about becoming incontinent, or losing control of my pouch because of fistula surgery | 1 |
| I worry that I may need to have more surgery in the future for my fistula | 1 |
| I worry about getting another fistula or abscess | 1 |
| I worry about needing a stoma because of my fistula | 1 |
| I feel embarrassed about having a fistula | 0.87 |
| I feel sad/depressed because of my fistula | 1 |
| I worry about having children in the future as it may worsen my fistula symptoms | 0.87 |
| Symptoms from my fistula (e.g. pain/discharge) affect my sexual activity | 1 |
| Symptoms from my fistula affect my ability to get physically close/ be intimate with someone | 1 |
| How often has your sleep been disturbed because of your fistula? | 1 |
| How often is your ability to concentrate disturbed because of your fistula? | 0.75 |
| How often has the fistula limited your ability to work (household or work outside the home)? | 1 |
| How often has the fistula limited physical activities that you would like to do (swimming/exercise/running/cycling)? | 1 |
| How often does your fistula affect your choice of transportation (travelling by car, bus, train)? | 0.87 |
| I avoid attending social events because of my fistula | 0.87 |
| I have had to leave social events early because of my fistula | 0.87 |
| I only attend social events if I know there will be toilets nearby because of my fistula | 0.87 |
| I feel supported by my family and friends to manage my fistula | 1 |
| I worry about not being able to work, or needing to make changes to my job in the future due to my fistula (e.g. having to work from home) | 1 |

**Supplementary Table 3: PFA and factor loadings of PAVF -QoL-34 items following Varimax rotation (Kaiser normalization)**

| **PAVF -QoL** | **Symptoms** | **Discharge** | **Faecal incontinence** | **Future concerns** |
| --- | --- | --- | --- | --- |
| PQOL1= Do you get swelling around the anus/vagina because of your fistula? | .681 |  |  |  |
| PQOL2= How often do you experience discharge (pus/mucus/blood) from your fistula? |  | .439 | .389 |  |
| **PQOL3= Do you need to use pads/gauze because of discharge from your fistula?** |  | **.571** | **.513** |  |
| **PQOL4= How much pain have you had around your anus/vagina because of the fistula in the past 6 weeks?** | .**734** |  |  |  |
| PQOL5= How often do you experience discomfort from your fistula? | .716 |  |  |  |
| **PQOL6= Do you get skin irritation (chafing) because of discharge from your fistula**? | **.495** | **.473** |  |  |
| PQOL7= How often do you need to use medication for pain from your fistula? | .678 |  |  |  |
| PQOL8= How often have you felt unwell because of your fistula in the past 6 weeks? | .711 |  |  |  |
| **PQOL9= How often have you leaked stool through your fistula in the past 6 weeks?** |  | **.475** | **.590** | . |
| **PQOL10= How often do you use medication (e.g. Loperamide, codeine) to control leakage of stool through your fistula?** |  |  | **.548** |  |
| PQOL11= How often are you bothered by side effects of medications (loperamide, codeine, antibiotics, biologics, immunomodulators) used to treat your fistula? | .645 |  |  |  |
| PQOL12= I have to modify my diet to control the symptoms of my fistula |  |  | .723 |  |
| **PQOL13= I feel tired or drained because of my fistula** | **.657** |  |  |  |
| PQOL14= I am worried others may be able to smell the discharge from my fistula |  | .533 |  |  |
| PQOL15= I worry about what others might think as I need to frequently use the toilet because of my fistula | .465 |  | .415 |  |
| **PQOL16= I worry about becoming incontinent, or losing control of my pouch because of fistula surgery** |  |  |  | **.894** |
| PQOL17= I worry that I may need to have more surgery in the future for my fistula | .379 | .407 |  |  |
| **PQOL18= I worry about getting another fistula or abscess** | **.475** |  |  | **.466** |
| PQOL19= I worry about needing a stoma because of my fistula |  |  |  | .872 |
| **PQOL20= I feel embarrassed about having a fistula** | **.411** | **.512** |  |  |
| PQOL21= I feel sad/depressed because of my fistula | .541 | .550 |  |  |
| PQOL22= I worry about having children in the future as it may worsen my fistula symptoms | .546 |  |  |  |
| PQOL23= I worry about not being able to work, or needing to make changes to my job in the future due to my fistula (e.g. having to work from home) | .704 |  |  |  |
| **PQOL24= Symptoms from my fistula (e.g. pain/discharge) affect my sexual activity** | . | **.895** |  |  |
| PQOl25= Symptoms from my fistula affect my ability or desire to get physically close/ be intimate with someone |  | .909 |  |  |
| **PQOL26= How often has your sleep been disturbed because of your fistula?** | **.534** | **.418** | **.435** |  |
| PQOL27= How often is your ability to concentrate disturbed because of your fistula? | .734 |  |  |  |
| **PQOL28= How often has the fistula limited your ability to work (household or work outside the home)?** | **.809** |  |  |  |
| **PQOL29= How often has the fistula limited physical activities that you would like to do (swimming/exercise/running/cycling)?** | **.677** |  | **.381** |  |
| PQOL30= How often does your fistula affect your choice of transportation (travelling by car, bus, train)? | .654 |  | .421 |  |
| PQOl31= I avoid attending social events because of my fistula | .593 |  | .439 |  |
| **PQOL32= I have had to leave social events early because of my fistula** | **.584** |  | **.473** |  |
| PQOL 33= I only attend social events if I know there will be toilets nearby because of my fistula | .424 |  | .575 |  |
| PQOL34= I feel supported by my family and friends to manage my fistula | .043 | -.043 | -.152 | -.147 |
