# Supplementary material for: Capturing quality of life in pouch anal and vaginal fistula: development of PAVF-QoL-14, a patient-reported outcome measure
Source: Qual Life Res. 2026 Sep 3;35(10):279. doi: 10.1007/s11136-026-04380-z (PMC13541871; doi:10.1007/s11136-026-04380-z)
Supplement: Supplementary file 6 [file 11136_2026_4380_MOESM6_ESM.docx]

**Developing a pouch anal and vaginal fistula quality of life scale (PAVF-QoL)**

**Topic Guide for Semi-Structured Patient Interviews**

The interviewers will ask questions with the aim of better understanding the impact on quality of life of various conservative, medical and surgical treatments that they may have had. The interviews will enable us to understand what factors are perceived important from a patient perspective when deciding whether a particular treatment has been successful. Open-ended questions will be used to encourage free dialogue. This form of interview style will enable to us to develop a better understanding of patient views with regards to the impact of their treatment and their disease.

The following guide will be used as a tool to encourage conversation points specific to the assessment of quality of life.

The topic guide will most likely evolve with subsequent patient interviews as participants may raise important points of discussion not covered by the topic guide.

Participants will be encouraged to speak freely about aspects of the fistula that affect their lifestyle. The outline below will help the flow of the conversation especially if the participant requires prompting to discuss aspects of the disease that affect quality of life. It is unlikely that an interviewer will need to ask every question suggested under each point. The participant may need guidance for the discussion but should be enabled to speak freely.

1. **Introduction and consent**
a. Outline the purpose of the study
b. Ensure patient still willing to take part
c. Check if participant agrees that the interview be audio-recorded, stored and used for presenting research findings anonymously.
d. Check if there are any questions regarding the interview procedure
e. Ask participant to complete the informed consent form
2. **Explore patient experience of living with a pouch fistula**

*Ask about the experience of having a pouch anal or pouch vaginal fistula*

a. ‘I understand you have (had) a pouch anal/ pouch vaginal fistula. Can you tell me more about that?’
b. ‘How soon after your pouch was created did you notice symptoms which then turned out to be from a fistula?’/‘Could you tell me about how you first found out you had a fistula?’
c. ‘What type of symptoms did you have after being diagnosed with a fistula’/’ Did your pouch function deteriorate at the same time as the fistula developed?’
d. ‘Can you tell me what life with a fistula is like for you?’/’What is the impact on daily life?’/’Did it impact on your social life?’/’Did it impact on your sex life?’/’ Does it impact your work life?’
e. ‘What do you feel is the worst aspect of having a fistula?’
f. ‘How do you manage the fistula?’

3. **Ask about the treatment that was offered and how they decided about undergoing treatment**
a. ‘What treatment(s) were you offered, and which did you choose/ receive?’
b. ‘Were you aware of the pros and cons of the different treatment options?’
c. What made you decide on one treatment over the other?’/’What factors did you consider in deciding on a treatment?’
4. **Ask about the effects that treatment had/is having**
a. ‘Was your treatment successful, and why?’/’How did the treatment impact on daily life/ working life/ social life/sex life?’/’How did you notice that the fistula treatment worked (if it worked)?’/ ‘What factors are important to you in deciding whether a treatment has worked?’
b. ‘Did you experience any negative effects right after treatment or later on?’
c. ‘How bad were these negative effects? Would you choose this treatment again or recommend it to a family member?’
d. ‘What do you feel is the worst aspect of the treatment?’
e. ‘What would you say is the most important result after the treatment?’
f. ‘Did your perspective on what is important change over time?’
g. ‘Overall, were you satisfied with the results of the treatment? Did the treatment match your expectations?’

**5. What is the worse thing about living with a fistula?**

**It will be important to decipher symptoms that are specific to fistula and those specific to the pouch that affect quality of life.**

**Broad areas of discussion / aspects PVF can affect lifestyle:**

- General health

- Physical symptoms

- Daily activities

- Intimacy and relationships (initiating and maintaining)

- Sexual activity

- Social activities

- Hobbies / Physical exercise

- Working life

- Hygiene/ costs/ remedies

- Psychological aspect

- Sleep

- Stigmatization

- Worries about the future

- Emotional aspect

- Self perception

**Additional questions that may need to be asked:**

a. Were you adequately prepared and informed about your operation?
b. How was your post op recovery and follow-up experience?
c. What would you have liked to be done differently?
d. What has your experience been like with your surgeon?

**6. Have you ever filled in any questionnaires related to your fistula and if so, which one and how did you find the questions?**

**7. Show patient PDAI, HADS, SF12 questionnaires and ask an open ended question about their thoughts about the each.**

a. Do you think anything is missing in this questionnaires in the assessment of the impact of the fistula on your quality of life.

**8. Concluding remarks:**

- Thank you for speaking with me.
- Ask if there are any questions.
