# Supplementary material for: Capturing quality of life in pouch anal and vaginal fistula: development of PAVF-QoL-14, a patient-reported outcome measure
Source: Qual Life Res. 2026 Sep 3;35(10):279. doi: 10.1007/s11136-026-04380-z (PMC13541871; doi:10.1007/s11136-026-04380-z)
Supplement: Supplementary file 7 [file 11136_2026_4380_MOESM4_ESM.docx]

**Supplementary Figure 1 Scree plot—PAVF -QoL-14 factor retention.**
